# Unmanned aerial vehicles (drones) in out-of-hospital-cardiac-arrest

**DOI:** 10.1186/s13049-016-0313-5

**Published:** 2016-10-12

**Authors:** A. Claesson, D. Fredman, L. Svensson, M. Ringh, J. Hollenberg, P. Nordberg, M. Rosenqvist, T. Djarv, S. Österberg, J. Lennartsson, Y. Ban

**Affiliations:** 1 Department of Medicine, Karolinska Institutet, Solna Center for Resuscitation Science, Stockholm, Sweden; 2 Department of Clinical Science, Karolinska Institutet, Danderyd University Hospital, Stockholm, Sweden; 3 Department of urban planning and environment, division of geoinformatics, The Royal institute of technology (KTH), school of architecture and the built environment, Stockholm, Sweden

**Keywords:** Cardiac arrest, EMS, Defibrillation, AED, UAV, Drone

## Abstract

**Background:**

The use of an automated external defibrillator (AED) prior to EMS arrival can increase 30-day survival in out-of-hospital cardiac arrest (OHCA) significantly. Drones or unmanned aerial vehicles (UAV) can fly with high velocity and potentially transport devices such as AEDs to the site of OHCAs. The aim of this explorative study was to investigate the feasibility of a drone system in decreasing response time and delivering an AED.

**Methods:**

Data of Global Positioning System (GPS) coordinates from historical OHCA in Stockholm County was used in a model using a Geographic Information System (GIS) to find suitable placements and visualize response times for the use of an AED equipped drone. Two different geographical models, urban and rural, were calculated using a multi-criteria evaluation (MCE) model. Test-flights with an AED were performed on these locations in rural areas.

**Results:**

In total, based on 3,165 retrospective OHCAs in Stockholm County between 2006–2013, twenty locations were identified for the potential placement of a drone.

In a GIS-simulated model of urban OHCA, the drone arrived before EMS in 32 % of cases, and the mean amount of time saved was 1.5 min. In rural OHCA the drone arrived before EMS in 93 % of cases with a mean amount of time saved of 19 min. In these rural locations during (*n* = 13) test flights, latch-release of the AED from low altitude (3–4 m) or landing the drone on flat ground were the safest ways to deliver an AED to the bystander and were superior to parachute release.

**Discussion:**

The difference in response time for EMS between urban and rural areas is substantial, as is the possible amount of time saved using this UAV-system. However, yet another technical device needs to fit into the chain of survival. We know nothing of how productive or even counterproductive this system might be in clinical reality.

**Conclusions:**

To use drones in rural areas to deliver an AED in OHCA may be safe and feasible. Suitable placement of drone systems can be designed by using GIS models. The use of an AED equipped drone may have the potential to reduce time to defibrillation in OHCA.

## Background

Out-of-hospital-cardiac-arrest (OHCA) is one of the leading causes of death in Europe, affecting about 300,000 people annually [1].

Emergency medical services (EMS) in Sweden report approximately 5,000 cases of OHCA each year to the Swedish registry for cardio-pulmonary resuscitation (SRCR) in which cardio-pulmonary resuscitation (CPR) was initiated [2]. It has previously been shown that early defibrillation in OHCA increases survival. Nevertheless, in rural areas there is usually an increase in distance due to prolonged response time for EMS and thereby a delay to first shock, which has a direct negative association to survival [3].

Dual dispatch using fire departments or police has been shown to shorten the response time and increase survival; however, the effect on 30-day survival is most significant in urban or downtown areas as compared to rural. Bringing an AED to the scene within its first minutes can dramatically increase survival [4].

A novel way of decreasing the delay from collapse to first shock in areas with long EMS response time could be to use a drone equipped with an AED. Drones may increasingly be used in the future by EMS for delivery of medical equipment, in major incident situations or for video surveillance [5, 6].

Simulation studies have found that the use of drones in emergency settings is most efficient and effective when flown on auto-pilot as compared to manual navigation [7]. Limitations in wind, flight endurance, payload and regulations need to be ensured for safe drone usage [8].

There is a large potential for many possibilities to use drone systems to transport and deliver an AED in cases of OHCA. The aim of this explorative study was to describe the potential benefit of a drone system to decrease the response time in OHCA in two different theoretical models. The second aim was to investigate the practical use of a drone for delivering an AED applied on historical OHCA, i.e. to describe safety and efficacy by using this kind of new system.

## Methods

The analysis for this study consisted of two main subsections: analysis of suitable drone placement using GIS-models and delivery test-flights on these sites with a UAV system.

### Stockholm County

This explorative study was carried out in Stockholm County, Sweden, which covers a total area of 6,488 km^2^ and has a population of 2,224,156 inhabitants, producing an average density of 343 individuals/km^2^ [9] The county consists of both rural areas with <250 inhabitants/km^2^ and downtown areas in the city centre with ≥6000 inhabitants/km^2^ [4].

The incidence of OHCA in Stockholm County is 46/100,000 per year. Four dispatch centres receive emergency “112” calls originating in Stockholm County and dispatch 58 ambulances during daytime and 38 at night. The EMS operates a two-tier system providing ALS treatment and is staffed with mainly registered nurses with university paramedic training. Dual dispatch parallel to EMS dispatch is carried out by police and fire departments [10].

In OHCA cases in Stockholm County presenting with a shockable rhythm, the median response time from collapse to defibrillation was 11 min, and survival to 30 days was 31 % for EMS cases versus a 70 % survival rate if a public AED was used prior to EMS arrival [10].

### Analysis of optimal drone placement using GIS-models

A spatial analysis of optimal drone placement was performed using geographic information system (GIS) tool ArcMap, and ArcGIS 10 [11] was used to analyse and visualize the results. Each area on a raster layer covering a map of Stockholm County was given a value based on EMS delay and incidence of OHCA.

This raster with interpolated values was produced from EMS delay times and was weighed against a raster created from the density of previous validated non-crew witnessed OHCA cases with a presumed cardiac etiology reported to SRCR in Stockholm County.

**Fig. 1:**
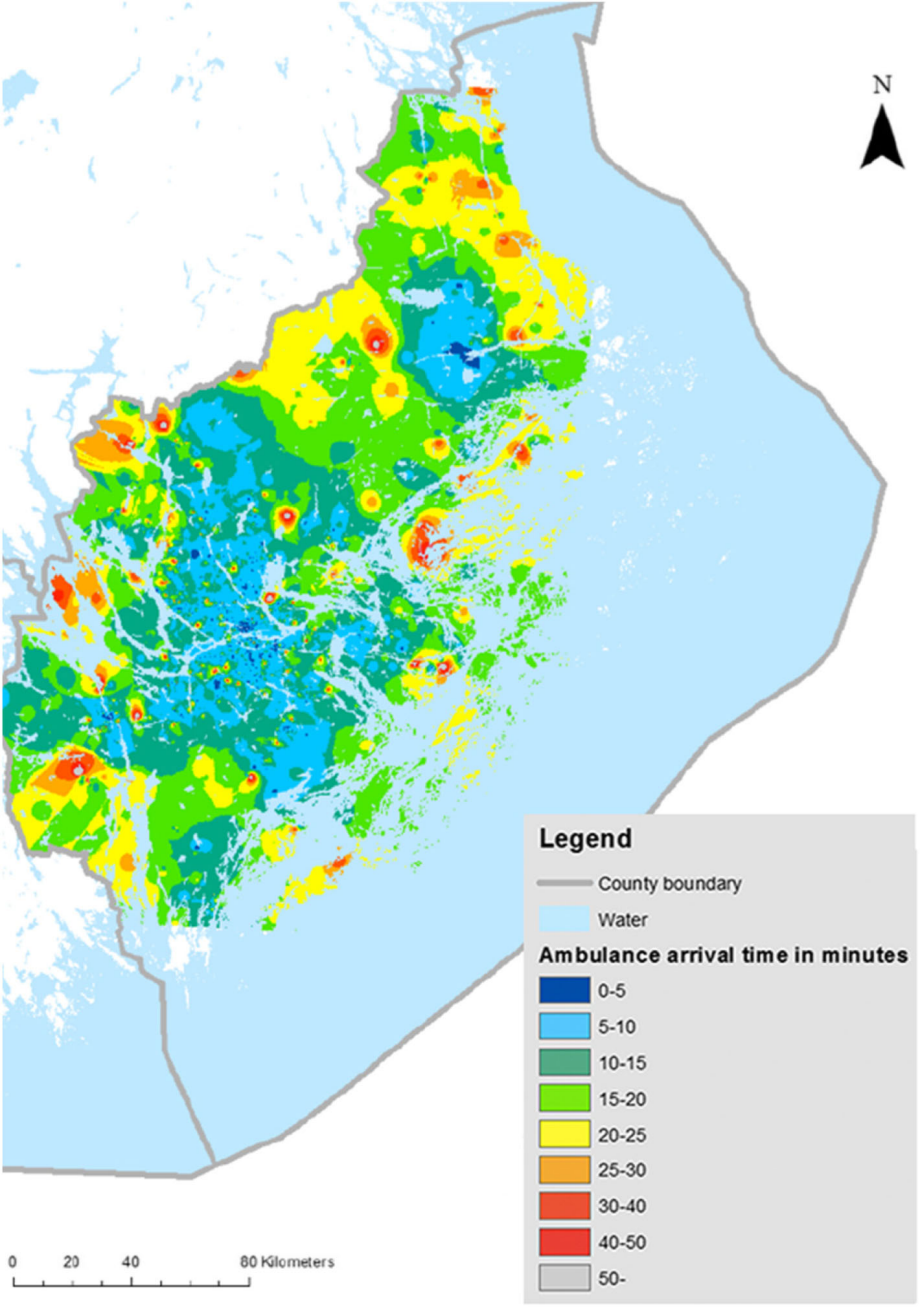
EMS response time in OHCA, Stockholm County 2006–2013. Ambulance arrival time in minutes, Stockholm County 2006–2013. Non-crew witnessed, cardiac etiology, *n* = 4,385 cases

A Point Density (PD) tool counted the number of OHCA within several distinct areas providing a raster layer giving values representing the density. Inverse distance weight interpolation (IWD) is an additional tool that creates a raster from a point layer; it was used on the OHCA layer with EMS delay as input, Fig. 1.

Multi-criteria evaluation (MCE) is a spatial tool that was used to evaluate the most suitable placement of UAVs by integrating these different layers and ranking the importance of each layer. Every layer is multiplied by a weight which adds up to 1. Calculations of suitable drone placement were based upon two alternative scenarios.

### Urban locations–50/50 weighting, Fig. 2

**Fig. 2:**
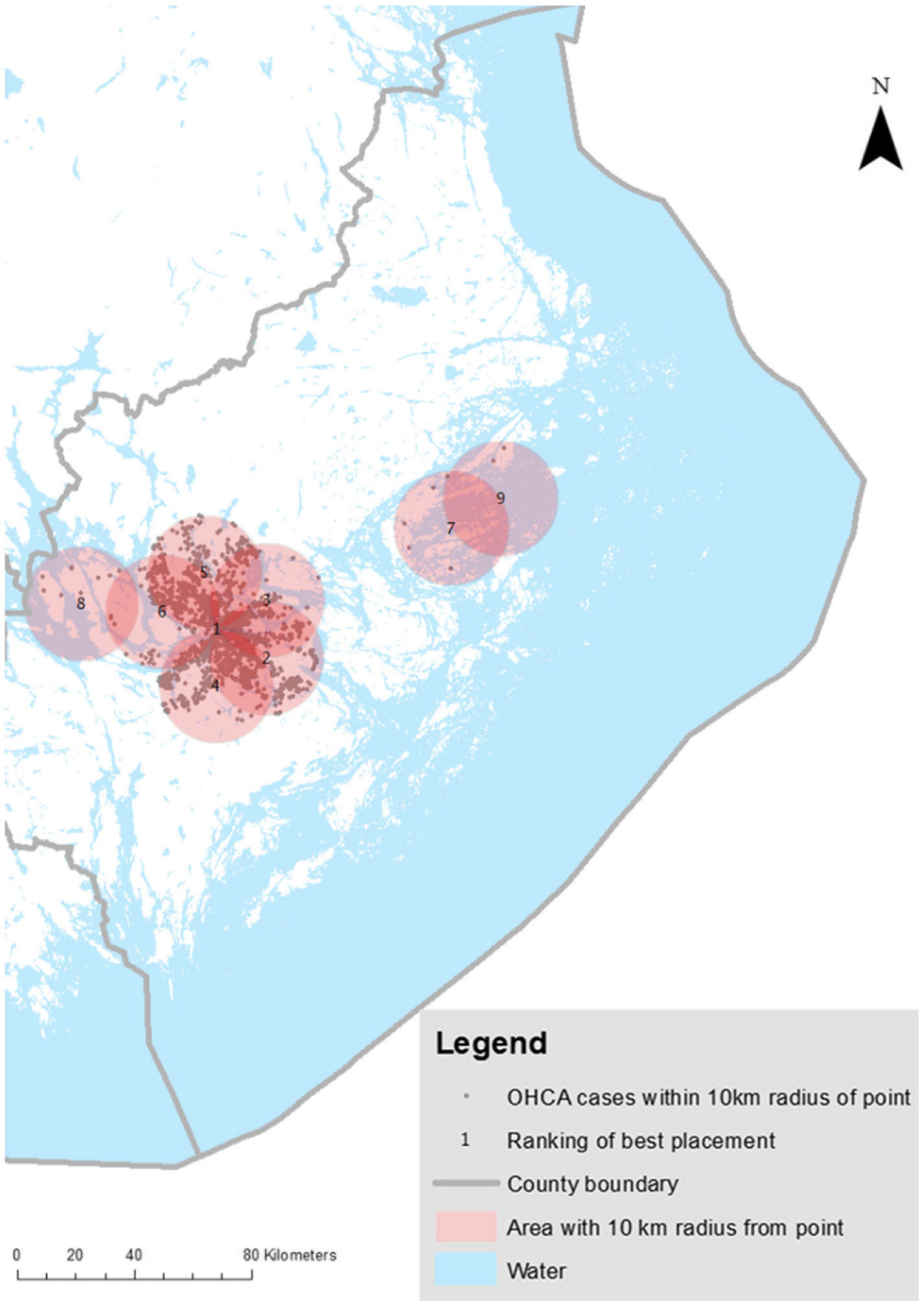
Suitable placement of UAV in an urban setting using a 50/50 weighting. Optimal placement of UAV, using a 50/50 weighting alternative. OHCA cases *n* = 3,041 between 2006–2013 in Stockholm County within a 10 km radius of point from optimal placement of UAV. Location #10 coincides with location #1 and was therefore excluded from visualisation in this figure

In order to find suitable placement for the drones, this MCE model favoured EMS delay and OHCA incidence equally and gave these factors 0.5 weight points each.

### Rural locations–80/20 weighting, Fig. 3

**Fig. 3:**
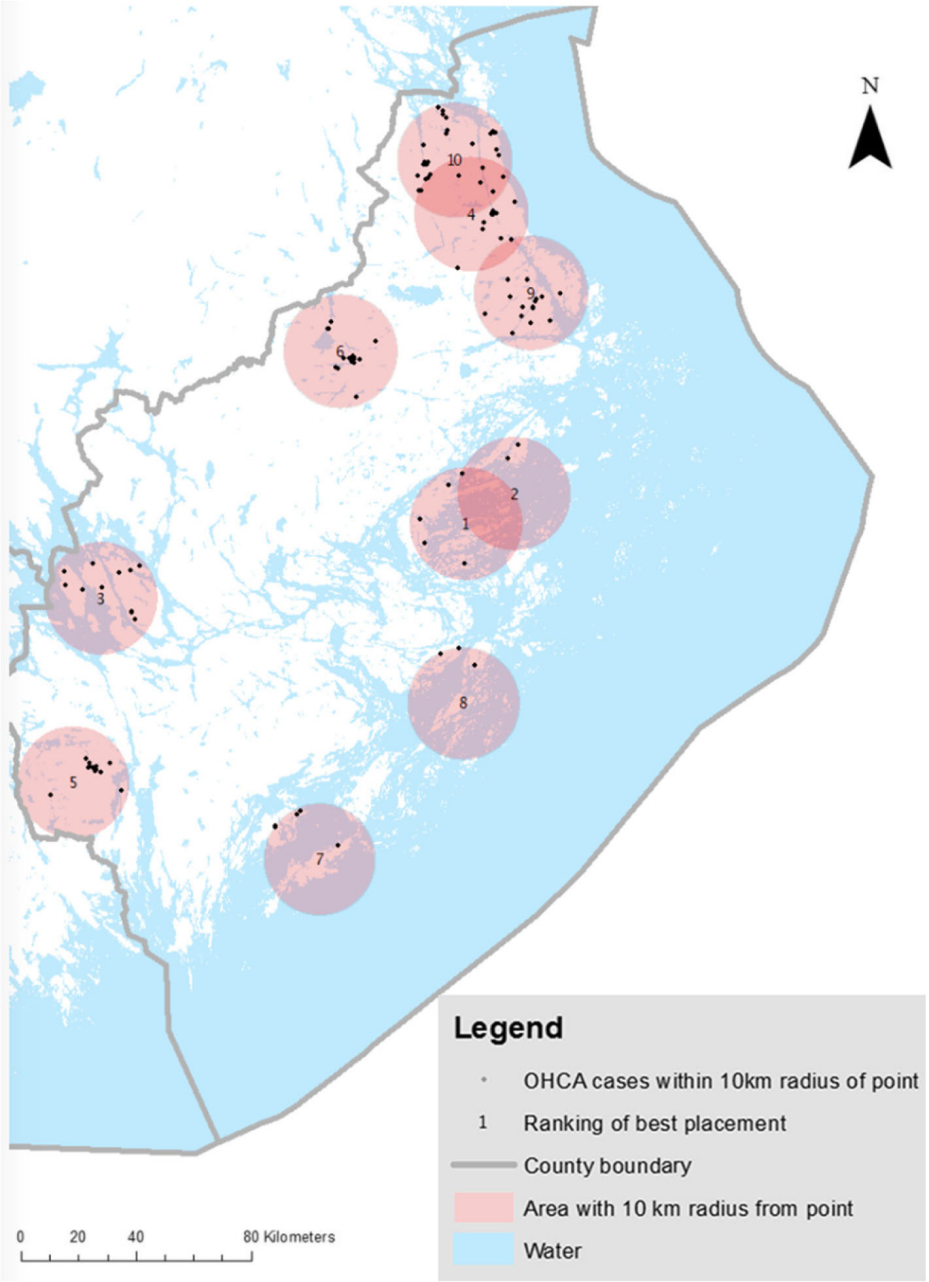
Suitable placement of UAV in rural setting using an 80/20 weighting. Optimal placement of UAV, using an 80/20 weighting alternative. OHCA cases *n* = 124 between 2006–2013 in Stockholm County within 10 km radius of point from optimal placement of UAV

In order to find suitable placement for the drones, this MCE model gave 0.8 weight points to EMS delay and 0.2 weight points to OHCA incidence, favouring a more extensive EMS delay in addition to a presumed low OHCA incidence.

Each circle on the map was given a 10 km radius from the suitable location, equalling an 8.5 min UAV flight time (70 km/h).

### Test flights

In Sweden the use of drones by civilians is restricted; they can not be operated beyond a pilot’s range of sight [12]. Test flights within the pilot’s range of sight were therefore carried out in the rural areas calculated with data based on historical OHCA in the archipelago surrounding Stockholm County. Two different eight-rotor class 2 UAVs from HEIGHT TECH GmbH & Co. KG company (DE) were used. These were operated by two licensed UAV-pilots and flown in manual flight-command mode. The UAV had a maximum velocity capacity of 70 km/h, with a maximal range of 10 km. The drone was modified with two latches holding the AED in place which could be opened remotely by the pilot. The AED was also prepared with a small parachute which unfolded after the opening of the latch-release.

**Fig. 4:**
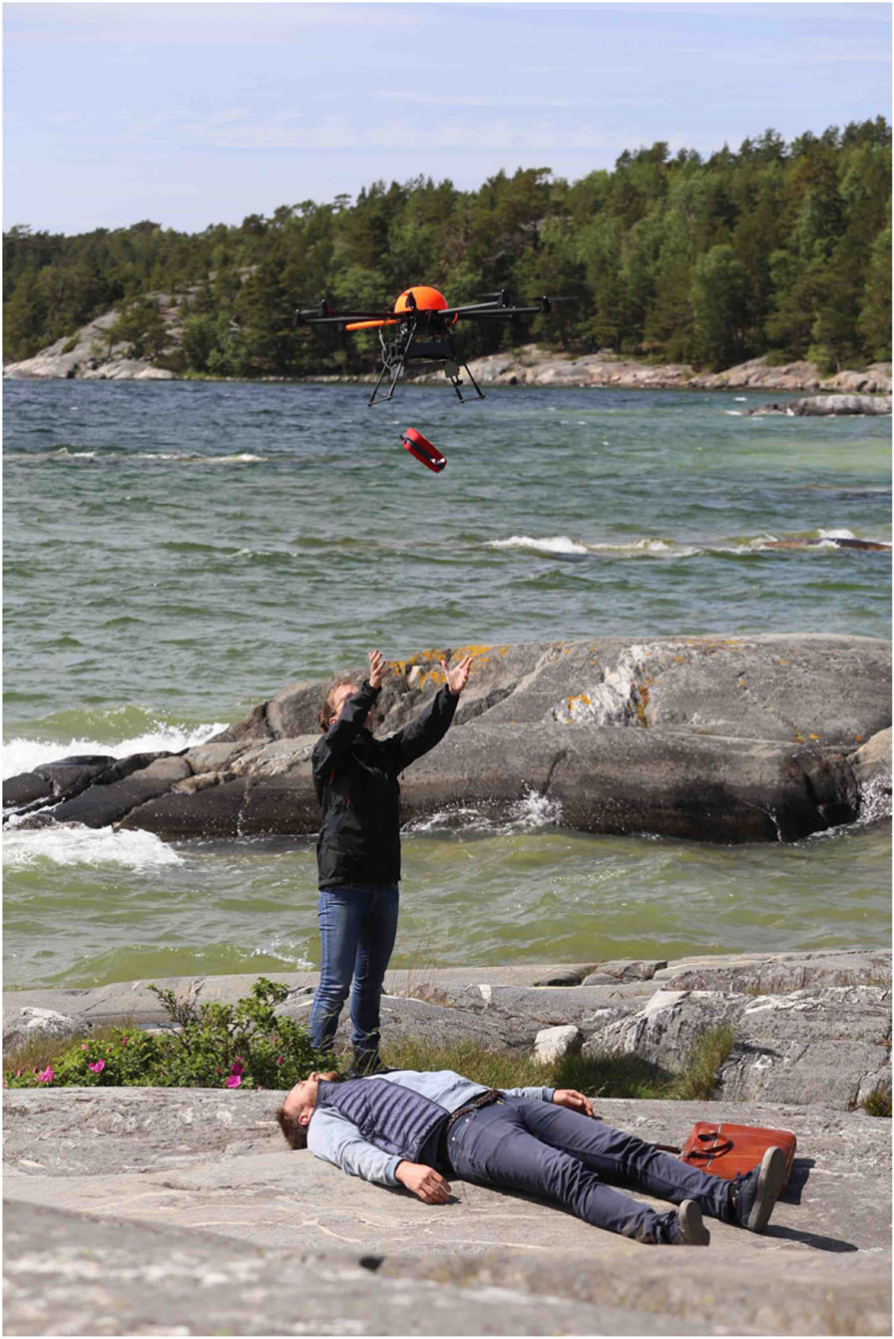
AED delivery using an UAV system. Delivery of an AED in simulated OHCA from 3 m altitude using latch-release from an UAV

For delivery of the AED, three different techniques were tested: (1) dropping the AED from the UAV using a parachute technique from high altitude, minimum 25 m, (2) dropping the AED from the UAV at an altitude of 3–4 m with a remote release system that included two latches holding the AED in place, Fig. 4, and (3) landing the UAV onsite.

Visual data on performance of the UAV was gathered in a structured protocol alongside meteorological data by the pilots and researchers. Basic performance of flying, hovering, delivery and landing on-site with an AED attached to the UAV was evaluated by the pilots and researchers after each flight. The AED was attached to a CPR-manikin post-delivery. Visual inspection was used to evaluate the AED alongside tests of functionality in terms of attaching and starting the AED.

### AED system

The AED, (Schiller AG - FRED easyport®) weight 490 g with additional supplement case had a total weight of 1 kg and was attached under the UAV.

## Results

### Finding the best suitable placement of UAV

**Fig. 5:**
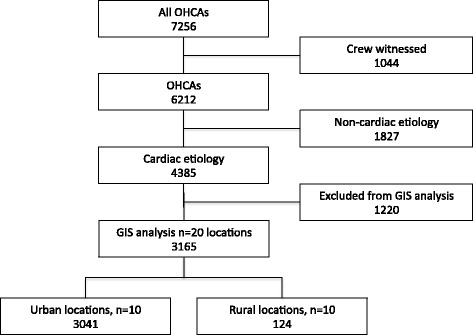
Flowchart of included cases. Flowchart of included cases. Final GIS analysis for optimal placement of UAV, *n* = 20 locations is based on non-crew witnessed cases with presumed cardiac etiology, *n* = 3,165 cases

A total of *n* = 7,256 OHCA cases were reported in Stockholm county between 2006–2013. Out of these *n* = 4,385 OHCA non-crew witnessed cases with presumed cardiac etiology were included in the theoretical GIS model and were available for analysis (see Fig. 5). In total, *n* = 20 suitable locations covering *n* = 3,165 cases (72 % of all cardiac OHCA) were identified by using this identification method. All OHCA cases where plotted within the reach of these twenty simulated UAV locations.

In urban cases (*n* = 3,041), 69 % of all cardiac OHCA with presumed shorter EMS delay and higher OHCA incidence, giving EMS delay time and OHCA incidence the same value (50/50 weighting), ten suitable locations for the UAV were found, primarily in the city centre (see Fig. 2). The UAVs were predicted to arrive before EMS in 32 % of OHCA cases, and the mean time saved by using a UAV was estimated to be 1.5 min (see Table 1).

**Table 1:** Potential of an UAV system for delivery of an AED in OHCA

Urban, 50/50 weighting	UAV, simulated maximum delay (min)	EMS, delay (min)^b^	UAV before EMS (min)^b^	UAV before EMS (%)
Location #:				
1 (471,1122)^a^	8,5	8,5 (0–93)	0	30 %
2 (368,864)	8,5	-	-	30 %
3 (250,710)	8,5	8 (0–93)	- 0,5	26 %
4 (323, 621)	8,5	9 (1–77)	0,5	34 %
5 (359,589)	8,5	9 (0–93)	0,5	39 %
6 (293,366)	8,5	10 (0–86)	1,5	44 %
7 (5,0)	8,5	31 (14–44)	22,5	100 %
8 (12,0)	8,5	24 (13–46)	15,5	100 %
9 (3,0)	8,5	32 (18–43)	23,5	100 %
10 (454,1095)	8,5	8 (0–76)	- 0,5	29 %
Total, (2538,5367)				32 %
Rural, 80/20 weighting	UAV, simulated maximum delay (min)	EMS, delay (min) ^b^	UAV timesaving (min)	UAV before EMS (%)
Location #:				
1 (5,0)	8,5	31 (14–44)	22,5	100 %
2 (3,0)	8,5	-	-	-
3 (12,0)	8,5	29 (13–46)	20,5	100 %
4 (21,0)	8,5	29 (19–43)	20,5	100 %
5 (14,1)	8,5	30 (11–81)	21,5	93 %
6 (23,1)	8,5	21 (11–62)	12,5	96 %
7 (4,1)	8,5	23 (9–40)	14,5	80 %
8 (3,0)	8,5	38 (6–82)	29,5	100 %
9 (15,1)	8,5	23 (5–41)	14,5	94 %
10 (24,6)	8,5	20 (3–54)	11,5	80 %
Total, (124,10)				93 %

^a^Numbers within parenthesis: (OHCA with UAV arrival before EMS vs OHCA with EMS arrival before UAV). Calculations based on suitable placements using a 50/50 vs an 80/20 weighting scenario, 8.5 min flight-time, UAV in 70 km/h velocity. Several cases are found within one or more UAV-locations, radius of each location 10 km

^b^ Mean delay (minutes) from call to arrival of EMS

In rural cases (*n* = 124), 3 % of all cardiac OHCA with longer EMS delay and low OHCA incidence, giving more value to a more extensive delay in EMS response time (80/20 weighting), ten other suitable locations for drone placement were found, all in remote areas (see Fig. 3). In this model, the UAV were predicted to arrive before EMS in 93 % of cases with a mean amount of time saved of 19 min (see Table 1).

### Drone delivery test flights

Manual test flights with AED equipped UAV (*n* = 13) were performed on recommended historical OHCA locations to evaluate the appearance of the UAV when carrying an AED (see Fig. 4). With the use of video link and flight data, pilots were able to safely control the UAV without disturbances in manoeuvrability.

### Drone delivery of the AED

Three different techniques for the drone to deliver the AED were tested. The best methods of delivering the AED were found to be the use of a latch-release from low altitude (3–4 m) and landing the UAV on flat ground. In delivering the AED on site, these were both safe for bystanders and superior to parachute release.

When using a parachute-release method (*n* = 1) wind-drift caused uncertainty about where the AED would land. When using a latch-release method (*n* = 6) at an altitude of 3–4 m, the bystander could fetch the AED as it released. The AED was fully functional and tested on a CPR-manikin (see Fig. 4). Landing the UAV (*n* = 6) on flat hard ground was a good alternative, in order to reduce risk for damage to bystanders eager to intervene, the rotors were shut off before bystanders approached the UAV. The AED was fully functional after landing on-site. No injuries were caused to bystanders, environment or to the drone itself.

## Discussion

This both theoretical and practical study explores a novel method for delivering an AED to the scene of an OHCA by using a drone. We calculated suitable locations for UAVs equipped with an AED in a major metropolitan area such as Stockholm county and in areas with substantial EMS delay. The use of an UAV in rural areas to deliver AED in OHCA may be safe and feasible. By using a GIS model [11] suitable placement of UAV systems can be designed and the use of an AED equipped UAV may potentially reduce time to defibrillation in OHCA.

Suitable placement of UAV and potential reduction in response time

With the use of data on EMS delay in response times as well as GPS-coordinates from retrospective OHCA cases, remote geographical areas can now be visualized. In remote areas with prolonged EMS response time (>20 min), the UAV might have real advantages in comparison to EMS in that a UAV may deliver an AED several minutes prior to EMS arrival. In contrast to our findings Pulver et al. estimated that a coverage of 80 % was met within one minute by placing drones at EMS-stations in an urban setting [13]. We believe this is optimistic and that the most significant time benefit will most certainly be found in rural areas, although the incidence of OHCA is less frequent than in urban areas. It is however important to take into account that a decrease in response time from ten minutes to seven minutes is less effective than one that goes from six minutes to three [4].

The difference in response time for EMS between urban and rural areas is substantial, as is the possible amount of time saved using this UAV-system. Changes in demographics over the year point out the need for a complement to EMS in rural areas. The suggested rural areas in this paper are largely inhabited in the summer time by people on vacation. Fire stations and sea-rescue stations in rural areas are reasonable alternatives for hosting such a system.

For implementation to be feasible, UAVs need to be implemented into the context of current jurisdiction, technological possibilities and existing search-and-rescue (SAR) infrastructure. Although time from call to dispatch has been set to zero for the UAV system, thus excluding time to recognition of the OHCA and dispatch, preliminary testing has shown that technical activation of the UAV (launch into the air) from the dispatch centre could be feasible within 10 s as compared to land-based EMS which can take up to 90 s in priority 1 cases. Helicopter emergency medical services (HEMS) which usually take up to 5 min before they are airborne. We believe that safety features, navigational planning and delay in seeking authorization from aviation authorities will be the main obstacles for this kind of system to be effective.

### Transportation of the AED

Previous testing of using UAV to transport medical products such as laboratory specimens has shown it is possible that the accuracy of samples can be completely unaffected by a test flight [14].

A change in legislation, implementation of a transponder, collision warning systems’ sound and lights, a delivery system, as well as a stable radio-link are all needed if a UAV is to be flown by automated means and out of a pilot’s visual range. Optimally UAVs should be deployed automatically with dual dispatch alongside EMS, and navigated via map-support. In commercial areas or in rescue scenarios, future autonomous flights can alleviate task interference and reduce the workload in the host/operating system [8]. An alternative to automated flights is to have a designated pilot requesting flight permission for an UAV that can be flown manually with a video-link.

### Delivery of the AED

During delivery of the AED onsite, we generally believe there is a risk the AED may be damaged when dropped to the ground or into an aquatic environment. Precautions needs to be taken in order to avoid causing harm to bystanders or the environment. The latch-release technique from 3–4 m height presents low risk of people being hurt from the rotors of the UAV. Adequate packaging of the AED may be needed, Fig. 4. Landing onsite is a preferable alternative for delivery. Optimally on flat ground using appropriate collision warning sensors as well as lights and sounds to attract attention. Bystanders onsite should be informed of incoming UAVs by the dispatch centre and instructed on how to locally enact appropriate safety measures. The dispatcher should not risk interrupting CPR; rather it should wait and inform the bystander once an AED is available in the vicinity. One could also consider deploying a drone in cases with two bystanders. Propellers should be shut off after landing, and AEDs should be placed on top of the drone, a more intuitive location for easy bystander access. Notwithstanding, modes of delivery need to be further evaluated in order to find a safe procedure for both AED and bystanders.

### Implementation

A majority of all OHCA cases with a presumed cardiac etiology present with ventricular fibrillation (VF) during the first minutes and early defibrillation is the key intervention. Non-cardiac cases may as well present with a shockable rhythm. [4, 10]

The rural cases can theoretically be reached by a UAV within 8.5 min from dispatch. Data from the SRCR suggest that 30-day survival rates in these cases can reach 30–41 % if defibrillation is carried out between 7–10 min, as compared to 0–8 % with an EMS delay of more than 21 min [2]. We believe that although the AED is not immediately present, the drone system can compensate for the EMS or HEMS delay.

The general public seems to have a neutral opinion regarding the risk involved with using UAVs; the risk is viewed as comparable to those of using existing manned aerial vehicles [15].

Implementing a new system such as this in addition to dual or even triple-dispatch in OHCA probably introduces new problems in the time-critical interaction between dispatcher, EMS and bystanders. Yet another technical device needs to fit into the chain of survival. We know nothing of how productive or even counterproductive this system might be in clinical reality. Current legislation today, however, restricts UAV flights for the purpose of delivering an AED that occur out of the range of pilots’ sight. Technical innovations and further studies on automated UAV-alert is needed to accurately deploy such a device with ensured safety and without delay.

Presuming that legal and technical requirements are met, we nevertheless believe that an autonomous dispatched UAV may have great potential in reducing time to first defibrillation.

## Limitations

We have only used data on OHCA from non-crew witnessed presumed cardiac etiology. Inclusion of non-cardiac cases would have resulted in more cases perhaps altering results. Data on UAV delays are simulations and not directly comparable to EMS response times as they lack time from call to dispatch and delay in landing procedures. There were missing data in 2 locations Urban 2 and Rural 2. However, calculations are based on the maximum UAV delay 8.5 min, in many cases delay would probably be shorter than shown here. For GIS analysis, weighting alternatives of 50/50 and 80/20 were used. Other weighting alternatives would have resulted in different suggested locations. As each UAV location covers a radius of 10 km, several OHCA cases in the analysis are overlapping. Data from a limited number of test flights regarding delivery of AED are the subjective experiences of the researchers and are not based upon quantitative data. The UAV used in test flights is just one of the many that are currently available and therefore perhaps may not be the most suitable. A different UAV system would have provided us with different conditions and perhaps altered results.

## Conclusions

To use drones in rural areas to deliver an AED in OHCA may be safe and feasible. By using GIS models suitable placement of drone systems can be designed. The use of an AED equipped drone may have the potential to reduce time to defibrillation in OHCA.

## Abbreviations

AED: Automated external defibrillator
CPR: Cardio-pulmonary resuscitation
EMS: Emergency medical services
GIS: Geographical information systems
GPS: Global positioning systems
IWD: Inverse distance weight interpolation
MCE: Multi-criteria evaluation
OHCA: Out-of-hospital cardiac arrest
PD: Point density
SAR: Search and rescue
SRCR: Swedish registry for cardio-pulmonary resuscitation
UAV: Unmanned aerial vehicle
VF: Ventricular fibrillation
